# An unusual presentation of necrotizing pneumonia caused by foreign body retention in a 20-month-old child: A case report and literature review

**DOI:** 10.3389/fped.2023.1203103

**Published:** 2023-06-15

**Authors:** Yuqi Wang, Yunlian Zhou, Feng Pan, Yingshuo Wang

**Affiliations:** ^1^ Department of Pulmonology, Children’s Hospital, Zhejiang University School of Medicine, National Clinical Research Center for Child Health, Hangzhou, China; ^2^ Department of Pediatrics, Xuancheng People’s Hospital, Xuancheng, China

**Keywords:** children, foreign body aspiration, necrotizing pneumonia, bronchiectasis, visit time

## Abstract

Necrotizing pneumonia (NP) is a rare but serious complication that occurs after foreign body retention. We report a case of severe NP in an infant caused by foreign body retention in the airway with no choking history. After a timely tracheoscopy and effective antibiotic treatment, her initial clinical symptoms were alleviated. However, she subsequently exhibited pulmonary manifestations of necrotizing pneumonia. To reduce the risk of NP from foreign body aspiration, for patients with airway obstruction and asymmetrical opacity of both lungs, timely diagnostic bronchoscopic evaluation is essential.

## 1. Introduction

Foreign body aspiration (FBA) is one of the most common accidents in children. It most frequently occurs when a young child is unsupervised, crying, or coughing. Complications of FBA include pneumonia, atelectasis, lung consolidation, pneumothorax, mediastinal emphysema, and rarely, necrotizing pneumonia, bronchiectasis, and death (1). Necrotizing pneumonia involves necrosis, liquefaction, and cavitation of the lung parenchyma due to infection. Currently, there are only two reported cases of necrotizing pneumonia caused by FBA (2, 3). Both are adult cases, and no cases in children have been reported. Poor outcomes of necrotic pneumonia can prolong a patient's disease course, impair lung function, and cause long-term impairment years after infection (4).

Shape, type, residence time, and other features of foreign bodies affect the likelihood of complications following their inhalation (5). If a guardian reports a history of foreign body inhalation, it is easy to identify the body in a child's respiratory tract. In contrast, in children without a clear history, the presence of an object may be missed, postponing diagnosis and treatment.

To emphasize the severe complication such as necrotizing pneumonia caused by foreign body aspiration in children, we report a case of severe necrotizing pneumonia caused by foreign body inhalation in a child with no apparent history of choking and negative multi-detector computed tomography (CT). This case also serves as a reminder that timely bronchoscopy can reduce foreign body retention time and provide benefits for patients.

## 2. Case description

**Figure 1:**
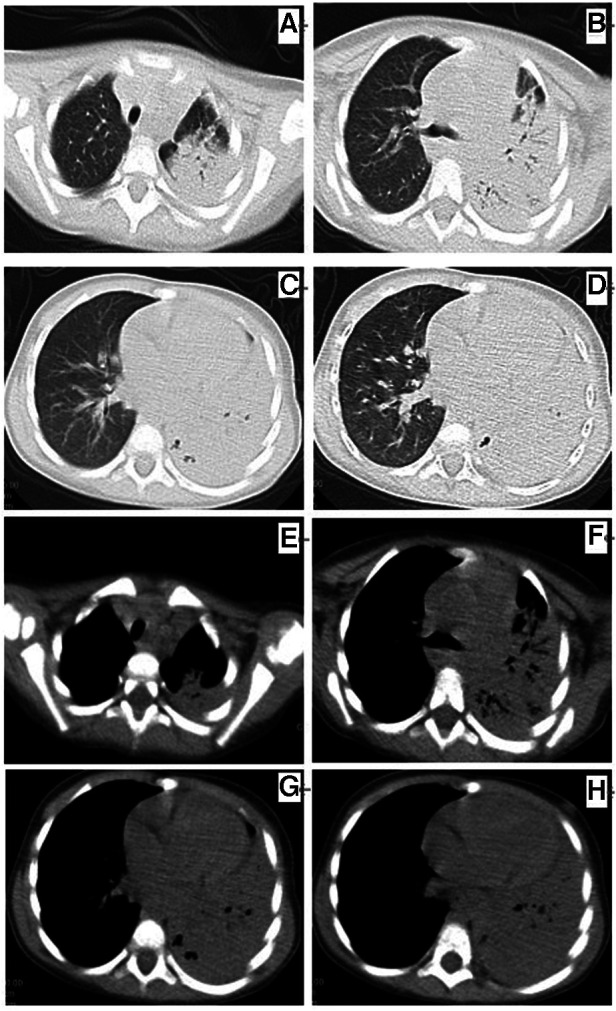
Chest plane computed tomography at 5 days before admission (**A–D**) showing left lung consolidation (lung window) and (**E–H**) consolidation of the left lung with a small amount of pleural effusion (mediastinal window).

A 20-month-old girl presented to our pulmonology department with chief complaints of cough for 1 month and fever for 2 weeks. She had a temperature of 38.0°C for 2 weeks. After experiencing cough for 3 weeks, she was taken to a local hospital. The patient's guardian denied any evidence of foreign body-associated choking or inhalation. Routine blood tests showed a white blood cell (WBC) count of 22.68 × 10^9^/L, neutrophil ratio of 69.2%, and C-reactive protein level of 75.88 mg/L. Chest plane CT showed consolidation of most of the left lung with insufficiency and a small amount of pleural effusion (Figure 1). Consequently, the patient received sodium cefotaxime for 5 days for the treatment of lung infection. Nonetheless, her symptoms did not diminish. Therefore, she was transferred to our hospital for further evaluation and treatment. Our treatment team works at a tertiary children's hospital, which is also a national regional medical center. Our department is also a training base for respiratory specialists, with more than 2,000 cases of children's flexible bronchoscopy performed every year.

**Figure 2:**
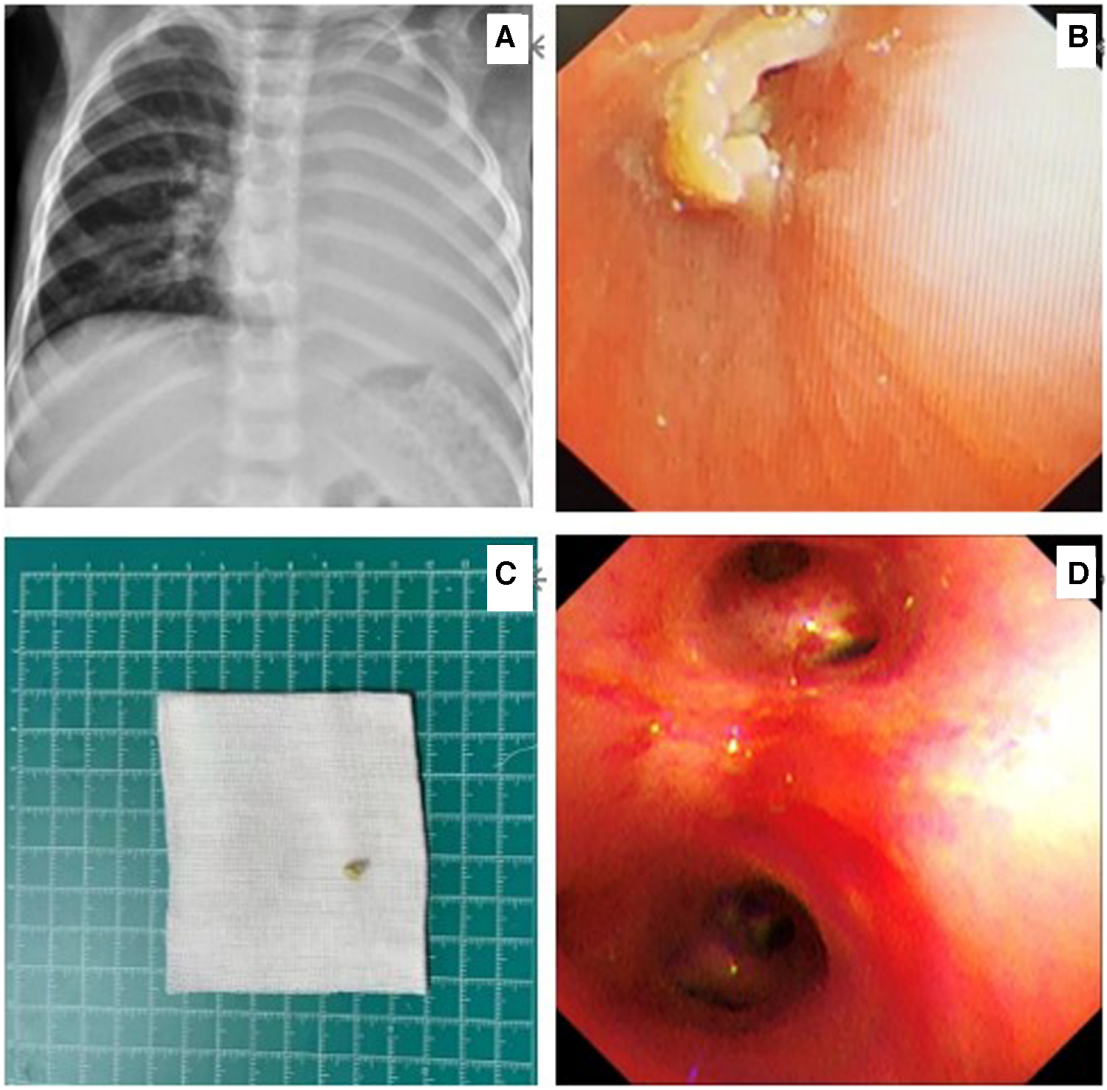
Chest x-ray on the day of admission showing atelectasis of the left lung (**A**). Flexible bronchoscopy showing a nut-like foreign body (**B**). A nut shell - like foreign object after removal (**C**). Review flexible bronchoscopy for foreign body retention (**D**).

When the patient was admitted to the hospital, she was in poor spirits and had shortness of breath and diminished breath sounds in her left lung. Her infection-related markers were significantly elevated. We treated her with intravenous antibiotics and performed an x-ray of her lung, which revealed opacification in the left lung indicative of pneumonia and left atelectasis (Figure 2A). Based on examination and imaging, she underwent flexible bronchoscopy and broncho-alveolar lavage. During the bronchial examination, an abnormal reflective object was found in the left main bronchus, which was removed using a foreign body retrieval basket (Boston Scientific Corporation M00513200), revealing a nutshell fragment (Figures 2B,C). Additional granulation tissue was observed around the original foreign body, with swollen mucosa that easily bled when touched. A large purulent discharge was released after removing the foreign body from the left main bronchus, and more granulation tissue formation around the original foreign body was observed. The broncho-alveolar lavage fluid (BALF) samples were assessed using metagenomic next-generation sequencing, which revealed 695 sequence reads associated with *Streptococcus pneumoniae*. Her BALF culture and blood culture results were negative. *Mycoplasma Pneumoniae* immunoglobulin M and immunoglobulin G and nucleic acid tests were negative.

**Figure 3:**
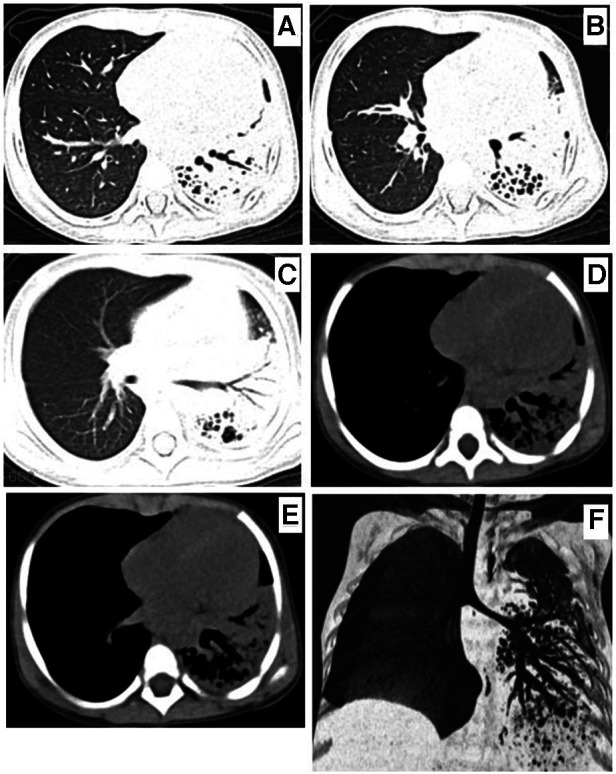
Chest computed tomography on the seventh day of admission showing left lung consolidation, atelectasis (**A–E**), and CT scan and airway reconstruction (**F**) multiple saccular changes in the left lung, indicating necrotizing pneumonia.

Enhanced CT for the lung on the seventh day of hospitalization showed infectious lesions in the left lung with consolidation, atelectasis, and multiple cystic changes in the lower lobe of the left lung, suggesting necrotizing pneumonia (Figure 3). She was treated with intravenous antibiotics of vancomycin (Day1–Day6) in combination with cefoperazone sulbactam sodium (Day1–Day14) and piperacillin tazobactam sodium (Day15–Day27) as anti-infective therapy.

**Figure 4:**
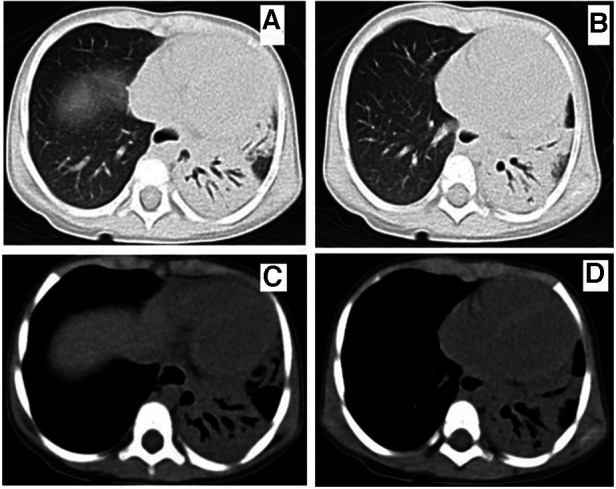
Chest computed tomography at 10 weeks of onset showed that bronchiectasis of the left lung was still present and the consolidation of left lung lesions was improved. **A-B** (lung window), **C-D** (mediastinal window).

Her temperature dropped to normal on the fourth day of hospitalization, but fever recurred between days 12 and 17 (peaking at 38.5°C) post-admission. No fever was observed from day 18 onward. She had less difficulty breathing and coughed less frequently. On her 18th day of hospitalization, a second flexible bronchoscopy was performed. Compared to the previous examination, there was a reduction in her left lumen secretions and the proliferative granulation tissue was clearly absorbed she was discharged on the 27th day of hospitalization and continued oral antibiotics for 2 weeks after discharge. A repeat CT performed after 2 weeks of discharge showed improvement in infectious changes in the left lung with pulmonary consolidation, atelectasis, bronchiectasis of the left lung, and cystic changes (Figure 4). She had no cough, fever, or any other clinical manifestations. We will continue to follow-up the patient for monitoring of cough, body temperature, dyspnea, exercise intolerance, chest CT changes, permissible lung function, and growth and development. The follow-up period will be further adjusted to ensure lung imaging recovery, lung function, and growth and development.

## 3. Discussion

Although bacterial infection of the lung due to foreign body retention is not uncommon, cases of necrotizing pneumonia resulting from this are rarely reported. Previous literature only reported two cases of necrotizing pneumonia in adults caused by foreign body aspiration (2, 3). FBA-related necrotizing pneumonia, a major complication, was found in a 20-month-old baby in this case. Her caregiver denied having a history of choking on a foreign body, and CT did not clearly show any evidence of the foreign body. However, tracheoscopy revealed the presence of a foreign body lingering in the left main bronchus, which was subsequently removed. The child developed a severe bacterial infection of the lungs and contracted a rare form of necrotizing pneumonia and bronchiectasis. This case serves as a reminder to pay attention to the timely diagnosis of foreign bodies in child's respiratory tract. The timing of bronchoscopy is crucial for diagnosing and treating the disease when pulmonary imaging displays obstructive airways or poor clinical efficacy.

Foreign body aspiration is a common cause of pediatric emergencies that can occur at any age, especially among children aged <3 years (6). The complication rate of respiratory foreign bodies in children is 16.1% (7). Secondary airway infection is a common complication of foreign body inhalation. Several studies have shown that the most common bacteria causing secondary bacterial infections after foreign body inhalation to the lower respiratory tract include *Streptococcus pneumoniae*, *Haemophilus influenzae*, and *Moraxella catarrhal*, while fungal infections are relatively rare (8). Infection with pathogens such as bacteria, fungi, and mycoplasma (9) can cause necrosis, liquefaction, and cavitation of lung parenchyma (10). The incidence of NP in children is approximately 3.2 cases per 10,000 cases (11). According to CT findings in this case, there were numerous cystoid alterations in the lower lobe of the left lung, lung consolidation, atelectasis, and infectious lesions in the left lung, suggesting necrotizing pneumonia. Based on the BALF's NGS test results, *Streptococcus pneumoniae* was identified as the causative pathogen.

In this case, the foreign body causing aspiration was a nut shell. In two previously reported adult cases, corn kernels (2) and conifer branch (3) were the foreign bodies. In a study of risk factors for lower respiratory tract infection caused by foreign body inhalation in children, inhalation of a plant, an object with an unsmooth surface, and a foreign body residence time of >7 days were independent risk factors for lower respiratory tract infection in children aged <2 years (12). The duration needed to identify a foreign body is strongly correlated with granulation formation, preoperative complications, and surgical and hospital stay duration (13). Compared to patients who visited within 2 days, those who waited longer than 2 days after aspiration were twice as likely to have complications. Additionally, compared to patients who received bronchoscopy within the first 2 h, those who received bronchoscopy 24 h or longer after arriving at the emergency department had a two-fold higher complication rate (14). According to a review of relevant literature, patients who aspirated for more than 72 h are at significantly increased risk of intraoperative and postoperative complications (9). The consultation rate within 1 week of symptom onset was 45.9%, while the consultation rates within 1 week to 1 month and longer than 1 month were 27% (15). The duration before medical examination and diagnosis time also affects the progression and prognosis of the disease (16). In this case, the patient's symptoms persisted for more than 1 month before treatment, which might have been one of the factors leading to severe pulmonary infection and necrotizing pneumonia. One possible explanation is that the parents lacked the necessary knowledge, did not pay sufficient attention to child care, and were unaware of potential risks.

Studies have shown that caregiver type; age of the child; level of education; number of children in the family; and knowledge, behavior, and attitude toward foreign bodies in the respiratory tract can influence the progression of disease caused by foreign body inhalation in children (17). Therefore, it is important to strengthen the publicity of foreign body knowledge, especially emphasizing the importance of seeking medical attention promptly.

Most patients develop cough symptoms after inhaling foreign bodies. Depending on the locations of the foreign bodies, patients may experience unique symptoms such as asphyxia and severe cough. Some foreign bodies may cause coughing or other obvious clinical symptoms for a period of time. Due to their prolonged presence, patients with foreign bodies in their respiratory tracts may develop symptoms such as fever and persistent cough. Foreign bodies in respiratory tracts of children are common. When a caregiver reports a history of foreign body inhalation in their child, the presence of a foreign body can usually be diagnosed easily. However, diagnosing foreign body inhalation in children without a clear history of illness can be challenging (18).

Pulmonary radiography is usually used to diagnose FBA (19). However, CT is not always sufficient for determining the presence or absence of foreign bodies. False-negative CT results have been associated with bronchial foreign body analysis. Foreign bodies in children are often hidden because caregivers cannot provide a clear history before bronchoscopy. Most patients who inhale foreign bodies are under the age of 3 years, and coughing and wheezing are primary symptoms. With this scenario in mind, the possibility of foreign body inhalation should be considered when assessing children. The presence of a foreign body in the respiratory tract should be considered, even in those with no history of aspiration, particularly when conservative treatment is ineffective, there is a history of foreign body inhalation but negative CT findings, or the patient has a chronic cough or recurrent respiratory disease. Previous research and clinical experience indicate that bronchoscopy is an important component of FBA diagnosis and treatment (19–21).

Necrotizing pneumonia is characterized by the destruction of the underlying lung parenchyma, resulting in multiple small, thin cavities. This can lead to more serious complications, such as bronchopleural fistula, empyema, respiratory failure, and septic shock, requiring closed thoracic drainage or even surgical treatment (4, 10). Compared to non-NP community-acquired pneumonia (CAP), patients with NP CAP account for higher resource utilization due to more complications; severe cases can even require mechanical ventilation (11). Our case of NP after FBA showed a long disease course. In this case, antibiotics were used for approximately 6 weeks, which is much longer than most CAP infections. The impact on the family's quality of life and financial pressures were obvious challenges due to the length of stay in hospital and the cost of treatment. After active treatment, the patient did not develop serious complications, such as bronchopleural fistula, empyema, and respiratory failure and shock. In the follow-up study of pediatric NP, patients still showed mild impairment of pulmonary function several years after onset, and pulmonary function might be impaired in adulthood (4). We will continue to follow-up our patient. CT of the child in this report indicated bronchiectasis at 7 weeks after the onset of the disease, and previous studies have also suggested that inhalation of foreign bodies is an important cause of bronchiectasis in children (22). Early tracheoscopy may prevent the risk of pulmonary resection (23).

Necrotizing pneumonia and bronchiectasis caused by foreign body inhalation are rare; only a few cases have been reported in the literature. Our case serves as a reminder that despite a lack of CT evidence or a history of foreign body inhalation, attention should be paid to the possibility of foreign body inhalation in patients with cough, fever, pulmonary imaging suggesting atelectasis, asymmetric opacity between both lungs, or prolonged drug therapy without lower respiratory tract improvement. Timely diagnostic bronchoscopic evaluation is essential. Children with NP, bronchiectasis, and other symptoms should be closely monitored to determine the cause of foreign body inhalation while they undergo active anti-infection therapy. Targeted treatment may improve the early active etiology and facilitate the identification of a lower respiratory etiology. The risks and outcomes of foreign body aspiration in children should be studied in the future. Educating the general public regarding this issue and close monitoring of children by caregivers is a preventive strategy. Therefore, education for childcare workers could be useful in preventing the occurrence of foreign body aspiration.

Since the follow-up time for this case was insufficient, we plan to monitor changes in lung imaging and their impact on her lung function in the future. For cases of severe complications caused by foreign body inhalation, such as NP and bronchiectasis, further studies are necessary to observe the disease development and its impact on children's lung function.

## Data Availability

The original contributions presented in the study are included in the article, further inquiries can be directed to the corresponding author.
